# Adenoid Vegetations of the Pharynx a Frequent Cause of Deafness in Children. Their Removal

**Published:** 1892-08

**Authors:** S. Latimer Phillips

**Affiliations:** Savannah, Ga.


### ADENOID VEGETATIONS OF THE PHARYNX A FREQUENT CAUSE OF DEAFNESS IN CHILDREN. THEIR REMOVAL.

BY

S. LATIMER PHILLIPS, M. D.,

Savannah, Ga.

It has been a matter of surprise to me upon looking over the text-books, in English and German, to find so little written upon this subject which of necessity must be of great interest to all physicians who deal much in the specialty of otology. In the last few years I have been lead to examine many cases which previously had been overlooked in this respect. Many cases which were diagnosed as simple catarrh of the middle ear, and treated as such, but with only partial success, were found to have their underlying cause in a growth in the pharynx, and after its extirpation got totally well.

It has become a matter of routine with me now to examine for this trouble the throats of all children brought to me to be treated for deafness. In a greater portion of these cases I find what I expect. It would almost seem, from the frequency of this trouble, that it was the cause of all catarrhal deafness in children, and if not the case, I believe but few can be found without more or less development of this nature in the pharynx.

A. H. Buck (in the 1889 edition of his Manual of Diseases of the Ear, page 175) confesses to having believed that the trouble was more prevalent in Europe than in this country until recently, having investigated the matter, and found it a very common one. He also gives here (l. c., page 171) a very good abstract from the original paper of Meyer upon this subject, published originally in Arch. für Ohrenheil., 1873 and 1874. Hartmann says that this condition is found in four per cent. of all aural affections. (Wilhelm Kirchner, Handbuch der Ohrenheil., page 97.) According to the observations of Hedinger, adenoid vegetations in the pharynx of children, with consecutive deafness, are more common from the age of three to ten years than any other. After the twentieth year they become very rare.

It is not necessary that the growth should be of such a large size as to interfere with breathing, to cause hardness of hearing, though in the great majority of cases, on inquiry, it will be found that the child sleeps with its mouth open and snores. The growth is often so large as to obstruct the pharyngeal openings of the nose altogether, thus causing mouth breathing and its train of unpleasant symptoms. What is more natural when such a growth can close the posterior openings of the nose into the throat, that even before it attains such a size it should infringe upon the walls of the Eustachian tube mouths, and thus shut off all circulation of air in the tympani? We have only to bear in mind that these adenoid vegetations are a hypertrophy of the membrane of the pharynx and its glands, and that this membrane extends up to the mouth of the tube, and is reflected on the interior as their lining, where it undergoes a change in its character.

Diagnosis can easily be made even if there are none of the attending symptoms, such as mouth breathing and snoring, by the introduction of the finger into the pharynx. One touch of the part by the experienced finger is sufficient. Instead of the smooth membrane which we find here in a normal condition, it will be elevated into folds or pleats, sometimes pedunculated enlargements the size of a cherry stone. These growths are usually found in the vault of the pharynx, but may be found on its lateral and posterior walls. On withdrawing the finger traces of blood on the end of it will generally be found. These enlargements are very vascular and the presence of blood a very good indication of the nature of the trouble. To make the examination I stand behind the chair of the child and press its head against my person, at the same time steadying it with my left hand under the chin; with a little coaxing the child is made to open the mouth, when the right index finger is passed into the mouth and up behind the soft palate and out again almost before the patient is aware that the examination has been made.

To illustrate this paper I will report two cases as follows: CASE I. Charles B., from Beaufort, South Carolina, aged 9 years. This little fellow has been under my treatment for several years for catarrhal deafness. When he was first brought to me was so deaf that he could scarcely hear a watch tick on contact. He was treated for a month with the Eustachian Catheter, Politzer's method of inflation, and application of arg. nit (gr. xxx to ℥i) to the pharynx almost daily. Under this line of treatment hearing improved materially, but it was only temporary, for whenever he had an access of cold deafness would return and he would have to go through the same treatment. He kept his mouth open all the time, as it was with the greatest difficulty he could breathe with it closed, and his mother complained of his making a great noise at night with his snoring, especially so after he had taken cold. An examination of the drum heads showed them sunken with loss of triangular light spot; they were freely movable by inflation. Over a year ago I examined his throat and found the pharyngeal space filled with adenoid growths. These were freely removed making a clear space through which he breathes now with ease. After the adenoids had been all cleared away the throat was sprayed out with an antiseptic solution and the tympanic cavities inflated. After a short treatment the hearing was almost completely restored. Since he has had no return of the trouble, and as far as I know, is perfectly well.

CASE II. H. M., of Thomasville, Ga., 9 years old, was under my treatment, three years since, for deafness. At that time the membranæ tympani were much sunken, and he had a chronic inflammation of pharynx. A diagnosis of chronic catarrh of the middle ear was made, and under the influence of inflations of the middle ear with Politzer's air bag and applications of arg. nit. to the pharynx he got much better. There was very great improvement in hearing and condition of membrane of throat. At times since he has suffered from attacks of earache accompanied by deafness which were attributed to cold. Last July he was brought back to me with history of an acute attack of earache, followed by deafness. The week previous could hear a watch tick only half inch away from each ear. On inquiry he was found to sleep with mouth open. On digital examination a quantity of growth was found in vault of pharynx, but not nearly so much as in the case reported above. In the usual manner this was all removed. After removal of growth hearing was completely restored, and he was enabled to sleep quietly with mouth shut. He has had one attack of earache since the growth was removed, but a couple of inflations with the air bag were sufficient to relieve him.

I could mention other similar cases, but the above are sufficient to answer my purpose.

The history of these cases will mostly be that the deafness developed rapidly, but I think such is not the case. The deafness comes on so gradually, *pari passu*, with the enlargement in the throat and the closure of the Eustachian tube, that it is not noticed until quite far advanced; then parents are so apt to mistake any sign of deafness in children for inattention. I have known them repeatedly to scold the little ones and sometimes even to go so far as to whip them for what they considered inattention, which after an examination was found to be deafness. Children are naturally bright and quick, and when one who previously has appeared so becomes dull and inattentive the ears should be looked after. As soon as any deafness in a child is observed, however slight, it should be attended to without delay. The recognition of the nature of the trouble and its proper treatment may save the child long years of suffering, and what may become later on a form of incurable deafness. In most of these cases of adenoid growth, its removal will result in perfect recovery from the deafness, as the growths do not recur. An early and prompt removal is always advised.

Having made sure that the growth is there the next thing to do is to remove it. This will have to be done by mechanical methods, as no sprays or acids will, in my estimation, be of avail. A number of measures have been devised by different writers to remove these growths. I presume all are good in the hands of those who are familiar with their use. Some employ a ring-knife, using the finger to locate the growth; others have a metal shield made to fit over the index finger, with which they go into the pharynx and scrape away the vegetations; still others claim to employ the cold snare by introducing it through the nose, engaging the growth in the wire loop and then drawing it home.

Many operate by whatever method with patient under the influence of an anæsthetic and the whole growth recovered at one sitting. I do not think it at all necessary to give an anæsthetic, and believe it safer to do the operation at several sittings than at one. Children at all reasonable upon finding there will be little pain will, after the first or second attempt, keep quiet and have the operation completed. Those younger and harder to manage will of course have to have the mouth-gag applied. The operation I do is the following: Placing the child upon a chair or the lap of the mother or attendant, with back to a bright window, I sit facing it and hold the tongue down with a depressor in left hand; having the throat well lighted now, I pass up behind the soft palate Gleichman's forceps, which were devised for this operation, with beaks closed; it is forced well up into the vault of the pharynx, and when it is felt to be touching something soft and spongy, the blades are opened and whatever is there grasped between them and withdrawn; the sharp edges of the blades easily cutting it off from its attachment; if there should be much resistance the blades should be opened, the growth let loose and the forceps withdrawn, and when reintroduced a smaller quantity seized; undue force should never be employed. The operations should be kept up until the walls of the pharynx are smooth to the finger, which should be introduced after every operation, to smooth over any roughness. The intervals between operations should be gauged according to size of growth and condition of parts after each operation. Bleeding is trifling; never have I seen it in any quantity. Pain is slight. Previous to an operation cocaine may be sprayed into the throat behind the palate. In the majority of cases I do not use it. It is well, after operations, to spray pharynx with some antiseptic solution.

The advantages of the above operation are:

1st. The doing away with all anæsthesia and its consequent dangers to life, such as sucking blood into windpipe, heart failure, etc.

2d. Ease with which it is done and its comparative freedom from pain and lengthy manipulation in pharynx.

3d. Ease with which the consent of the parents is obtained without anæsthesia.

